# Lessons and challenges of practice of traditional medicines as an alternative for COVID-19 vaccine in Tanzania

**DOI:** 10.4102/jphia.v16i3.708

**Published:** 2025-04-18

**Authors:** Paul E. Kazyoba, Chima E. Onuekwe, Alexander Makulilo, Tumaini Haonga, William Mwengee, Grace Saguti

**Affiliations:** 1 Department of Research and Development, Mabibo Traditional Medicine Research Centre, National Institute for Medical Research, Dar es Salaam, United Republic of Tanzania; 2 Department of Immunizations, Emergency Preparedness and Response (EPR), World Health Organization, Dar es Salaam, United Republic of Tanzania; 3 Centre for Health and Allied Legal and Demographical Development, Research and Training (CHALADDRAT), Nnamdi Azikiwe University, Awka, Nigeria; 4 Department of Political Science and Public Administration, Faculty of Social Sciences, University of Dar es Salaam, Dar es Salaam, United Republic of Tanzania; 5 Department Preventive Services, Health Promotion Unit, Ministry of Health, Dodoma, United Republic of Tanzania; 6 Department of Emergency Preparedness and Response, World Health Organization, Dar es Salaam, United Republic of Tanzania

**Keywords:** traditional, medicines, COVID-19 vaccine, political influence, Tanzania

## Abstract

**Background:**

The COVID-19 pandemic brought a peculiar experience across the world as populations were engulfed in shocks and panic because of lack of substantive interventions during its early days. Different intervention options were tested, including traditional medicines. Despite the lack of scientific evidence on safety and efficacy, traditional medicines brought calmness and dispelled fear associated with the disease in Tanzania. On the other hand, the introduction of COVID-19 vaccines was met by hesitancy, rumours and doubts about its safety and efficacy.

**Aim:**

This study aimed at understanding how promotion of traditional medicines during the early days of the pandemic affected timely introduction and uptake of COVID-19 vaccines.

**Settings:**

The study was conducted in a mix of urban and rural districts, purposively sampled from eight zones of the country.

**Methods:**

This was a cross-sectional study which employed the qualitative methods including key informant interviews and focus group discussions in eight zones of Tanzania.

**Results:**

Political advocacy on the use of traditional medicines in treating COVID-19 received a massive response across the country. This was driven by a lack of modern medicines during the early days of the COVID-19 pandemic outbreak. The introduction and promotion of COVID-19 vaccines in Tanzania provides a learning experience for future epidemics.

**Conclusion:**

Political advocacy influenced public leaning towards traditional medicines, while creating hesitancy on COVID-19 vaccines in Tanzania. This experience emphasises on using scientific evidence to promote interventions during health emergencies.

**Contribution:**

Research on traditional medicines focusing on infectious diseases outbreaks is emphasised to generate evidence which will guide advocacy on its use.

## Introduction

Traditional medicine commands a fair influence and presence in the community health system and contributes to over 60% of the primary healthcare services in the developing countries.^1^ In Tanzania, it is estimated that over 70% of the population uses traditional medicine in the treatment and management of diseases.^2^ The COVID-19 outbreak brought a peculiar experience across the world, as populations were engulfed in shocks and panic, particularly when the outbreak was declared by the WHO as a pandemic.^3^ The virus was spreading at an alarming speed with no reliable interventions available during the early days.^3^ The highly contagious nature of the COVID-19 virus heightened the demand for alternative interventions especially in Africa where the health systems are weak and burdened by other infectious diseases.^4^ There was no clear treatment plan from modern medicine at that time apart from treating symptoms of the infection. Evidently, with such a transmission rate and lack of modern medicines coupled with an increasing fear and uncertainty, people had to go their own ways to find the cure and prevention against COVID-19.^5^ In Tanzania, the government urged citizens to turn to traditional medicines and prayers according to each one’s religion as interventions against COVID-19.^4^ Strategically, this was done to quell public fear and panic because of lack of medications for COVID-19. It further provided psychosocial stability and public cohesion in the search for medical solutions against COVID-19.^4^

During the early days of the COVID-19 pandemic, leaning towards traditional medicines as an alternative to unavailable modern medicines raised concerns on safety and efficacy.^6^ The WHO urged African governments to ensure there is credible scientific evidence on safety and efficacy to support the use of traditional medicines in the treatment of COVID-19.^3,6^ The WHO statement came out after the Madagascan government claimed that COVID Organic (CVO) was a safe and efficacious herbal formula for the treatment of COVID-19.^7^ Many countries including Tanzania quickly sought to look for CVO in Madagascar. This development in Madagascar encouraged the population to continue leaning towards traditional medicines as the available solution for COVID-19.^8^ This raised interests among traditional health practitioners (THPs), herbalists and the population in general to trust traditional medicines as part of the interventions for COVID-19 infection. The impact of this was the emergence of several innovations on herbal medicines ranging from oral formulations to steam inhalation products. Some of these include NIMRCAF^©^, covidol, pupiji, uzima, shengena, covotanxa, planet++ and Bingwa, among others.^9^ Further efforts to promote traditional medicine included the fabrication and installation of steam inhalation chambers at some prominent public hospitals in the commercial city of Dar es Salaam.^10^ Again, this was an attestation of how policy direction influenced and promoted traditional medicines by getting deeper into the health system and lives of the people across the country.

When the development of COVID-19 vaccines became a reality followed by a speedy rolling out in different countries in early 2021, Tanzania accepted this reality with cautions and doubts, sighting safety concerns of the vaccines.^11^ Additionally, the negative rumours on safety and the myth fomented the level of doubts and mistrust of COVID-19 vaccines among populations, despite increasing scientific evidence on their effectiveness.^12,13^ With the policy and decision-makers raising doubts, the populations across the country were left confused and even more scared of COVID-19 vaccines.^13^ This largely had a negative impact on the timely introduction and acceptance of COVID-19 vaccines in the country.^13^ Various researches on COVID-19 vaccines rollout in Africa have reported challenges faced in the uptake of vaccines. The challenges were grouped into three factors: political, health system and community.^14,15,16^ For Tanzania, the country needed a new political direction addressing the three factors and introduction of COVID-19 vaccines^9^ in the subsequent response strategies.

While the three factors had a clear influence on the uptake of COVID-19 vaccines, a deep entrenched trust on traditional medicines and nutraceutical uses, which was backed by the political environment, had a substantial influence on the early acceptability of COVID-19 vaccines. The fact that the traditional medicine gained overwhelming support and acceptance in Tanzania does not mean that it was always a smooth alternative. It is unclear what challenges it encountered as an alternative to the COVID-19 vaccine, and this is one of lessons that should be learnt from such scenarios.

This article describes the lessons learnt and challenges of traditional medicine practices as alternatives to modern medicines during the early days of the COVID-19 pandemic in Tanzania. It further describes the political influence, which enhanced trust and leaning towards traditional medicines uses, consequently affecting the timely introduction and acceptance of COVID-19 vaccines. Lastly, this article describes the impact of political paradigm shift in changing the thinking and inclination of the population from traditional medicine to modern medicines (vaccines) without downplaying the contribution and importance of the former in the fight against COVID-19.

## Research methods and design

### Study design

This was a cross-sectional study that employed the qualitative methods by conducting key informant interviews (KIIs) and focus group discussions (FGD) in eight regions of Tanzania Mainland. The interview sought to determine in detail the traditional medicines and nutraceuticals used during the pandemic, and how this affected the uptake of COVID-19 vaccines. An additional data source is based on experience and review of response plans for COVID-19 and participation in the health-promotion activities during the COVID-19 pandemic.

### Study sites and study settings

The FGDs and KIIs were conducted from June 2023 to August 2023 in Arusha, Mbeya, Morogoro, Mtwara, Njombe, Shinyanga, Singida and Tabora. The regions were purposively sampled to represent the eight zones of Tanzania mainland. A mix of urban and rural district councils were purposively sampled informed by the rural–urban characteristics (Table 1).

**TABLE 1:** Regions and characteristics of councils covered during the survey.

S/N	Region	Councils	Urban/rural	Population (size as per 2022 census)
1	Arusha	Arumeru DC	Urban	331 603
		Karatu DC	Rural	280 454
		Arusha CC	Urban	449 518
2	Mbeya	Chunya DC	Rural	344 471
		Kyela DC	Rural	266 426
		Mbeya CC	Urban	371 259
3	Morogoro	Ifakara TC	Urban	290 424
		Morogoro DC	Rural	387 736
		Morogoro MC	Urban	471 409
4	Mtwara	Mtwara MC	Urban	146 772
		Mtwara DC	Rural	158 504
		Nanyamba TC	Urban	132 624
5	Njombe	Makete DC	Rural	109 160
		Njombe DC	Urban	109 311
		Makambako TC	Urban	146 481
6	Shinyanga	Kishapu DC	Rural	335 483
		Shinyanga MC	Urban	214 744
		Shinyanga DC	Rural	468 611
7	Singida	Ikungi DC	Rural	411 262
		Singida DC	Rural	284 895
		Singida MC	Urban	232 459
8	Tabora	Urambo DC	Rural	260 322
		Uyui DC	Rural	562 588

S/N, serial number; DC, District Council; TC, Town Council; CC, City Council.

### Data collection methods

#### Key informant interviews

The key informant’s interviews were conducted using pre-prepared interview guiding questions. The guiding questions were interpreted in Kiswahili for easy comprehension and response from respondents. A team of data collectors were trained on conducting interviews, particularly on how to probe for details on a particular subject. The KIIs involved political and religious leaders, leaders of various social groups such as Savings and Credit Co-operative Societies (SACCOS) and Motorbike driver’s associations, famous people in the communities, civil servants like teachers, nurses and clinicians, traditional birth attendants, THPs, traditional leaders and businessmen and women in the communities. Prior to engaging key informants, verbal consent was sought by explaining the scope and objective of the study, and then asking the respondents whether they agreed or declined to participate. The number of KII from each region depended on how fast saturation level was reached. A total of 340 KIIs were conducted across eight regions.

#### Focus group discussions

The FGDs were conducted using pre-prepared guiding questions to facilitate the discussions. The guiding questions were interpreted in Kiswahili for easy comprehension and response from respondents. Each FGD comprised of 8–12 participants aged 18 years and above who were drawn from the communities and had a wide knowledge of societal issues. Gender representation was considered while the representatives of different social groups within the community were also considered. Prior to engaging FGD participants, informed verbal consent was sought by explaining the scope and objective of the study, then asking participants whether they agree or do not agree to participate in the discussion. The total number of FGDs per site was determined based on the principle of saturation.^17^ Focus group discussions were recorded using audio recorders after seeking the consent of participants, and each session took a maximum of 1 h and 30 min. A total of 51 FGDs were conducted across eight regions covered during the study.

#### Document review

In order to complement the qualitative data collected from the field and provide a better understanding of the response strategies in Tanzania, the COVID-19 Response Plan Two was reviewed to establish the position of traditional medicine as a strategy used in the response against the pandemic.

### Data analysis (analysis of key informant interview and focus group discussions)

The qualitative coding and corresponding matrices were created and analysed using NVivo version 10.0. The software helped to identify potential correlations between different variables. Furthermore, descriptive statistics was used to determine the proportion of the population that used traditional medicines and nutraceuticals during the COVID-19 pandemic.

### Ethical considerations

Ethical clearance to conduct this study was obtained from the University of Dodoma, Institutional Research Review Ethics Committee (IRREC) (No. MA.84/261/76/214).

## Results

### Key informant’s interviews

A total of 340 key informants were interviewed across eight regions. Out of these, 65.3% were males and 34.7% were females. Results from KII have shown that 27.1% of all participants acknowledged using, or their community used, traditional medicines or nutraceuticals during the early days of the COVID-19 pandemic. Some acknowledged to have continued to use traditional medicines even when the COVID-19 vaccines were introduced in the country. Most participants cited indigenous knowledge (IK) and the political directives being the drivers for the people to overwhelmingly use traditional medicines particularly when modern therapeutics were not available. This was just like a struggle that was being experienced at the global level where people were desperately seeking therapeutics to treat and control COVID-19. The aggregated KII results on traditional medicines and nutraceutical uses are provided in Table 2.

**TABLE 2:** Key informant’s interviews: Response to the questions on what the community did whenever they had a patient with COVID-19 related symptoms?

Region	Number of KIIs conducted	Gender of KIs		Percentage of KI acknowledged to use, or their community used, TM and nutraceuticals
		Male	Female	
Arusha	81	45	36	28.4
Mbeya	37	25	12	40.5
Morogoro	38	21	17	21.1
Mtwara	18	15	3	38.9
Njombe	36	21	15	41.7
Shinyanga	29	21	8	20.7
Singida	82	57	25	19.5
Tabora	19	17	2	10.5

KIIs, key informant interviews; KI, key informants; TM, traditional medicines.

The utilisation of traditional medicines by communities to try to treat or prevent COVID-19 is evident across all regions covered in the study. Participants in Njombe and Mbeya regions had higher proportions of people who used traditional medicines and nutraceuticals (Table 2). Mtwara, Arusha, Morogoro, Shinyanga and Singida regions followed in that order, while Tabora registered a 10.5% usage.

### Focus group discussions

A total of 51 FGDs were conducted across the eight regions covered during the study. Out of these, 50.1% of the participants acknowledged the fact that their communities have used traditional medicines and/or nutraceuticals for the treatment or as a preventive strategy against COVID-19. It was further acknowledged that the practice was rampant during the early days of the pandemic because of the absence of vaccines and other therapeutics (Table 3) and health system issues. The most common practice was the steam inhalation. To do this, different regions used different combinations of herbs or leaves of food and medicinal plants species. Some of the food plants famously used at that time include leaves of the mango tree, guava tree and lemon and orange trees. The famous and common medicinal plants that were used for steam inhalation include neem tree, eucalyptus, menthol plant and some cloves. Some of the quotes from FGD participants attesting to this experience are as follows:

‘The government announced some interventions, for instance HE President urged citizens not to panic, as panicking is bad than the COVID-19 itself. He further urged citizen to resort to indigenous knowledge such as steam inhalation which has been used in traditional medicine to treat respiratory diseases. For us here we used a mixture leaf from different food and medicinal plants including eucalyptus, lemon tree, and guava tree. We also prepared mixtures of ginger, lemon, and garlic or onions which we provided to patients with COVID-19 symptoms.’ (FGD 3, PO7M Uyui DC, Tabora)

‘We did steam inhalation from neem plant leaves, and many people did steam inhalation. Fortunately, the leaves of a particular medicinal plant [*I have forgotten its name*] which was famously used in Dar es Salaam, are also used for the treatment of common colds and flu at our place. So, we have used steam inhalation to treat flu before, and it helps and completely heals you.’ (FGD 2, P10M, Chunya DC, Mbeya)

**TABLE 3:** Proportion of the population that acknowledged to have used traditional medicines and nutraceutical during the early days of the COVID-19 pandemic.

Region	Number of FGDs conducted	Percentage of FGDs that commented on using traditional and nutraceuticals (%)
Arusha	12	66.7
Mbeya	6	66.7
Morogoro	7	57.1
Mtwara	3	66.7
Njombe	6	83.3
Shinyanga	3	33.3
Singida	11	0.3
Tabora	3	33.3

Note: Factors that drove people to resort to TM and nutraceuticals: (1) Indigenous knowledge of herbs, traditionally used since time immemorial in the treatment of common colds and flu. (2) Government statements that became policy, leading people to heed the Head of State’s counsel.

FGDs, focus group discussions.

Additionally, there was a wide use of nutraceuticals, mostly those promoted by the government. This included a mixture of ginger, lemon, garlic and onions. Some added a small portion of cayenne pepper. This combination was initially developed by the National Institute for Medical Research (NIMR) in collaboration with the Traditional and Alternative Medicines Unit at the Ministry of Health. The combination of the five food stuffs, in a well-balanced formulation, was named as NIMRCAF^©^. Following the development, and high demand for this product, the formula was shared to the public so that it can be prepared at home. This helped citizens to prepare their own formulation at home.

When we compare the utilisation of traditional medicines across the eight covered regions, FGD participants from seven regions reported a higher utilisation of traditional medicines. The Njombe region had the highest proportion, with 83.3% of the FGD participants in the region confirming that they or their communities used traditional medicines and nutraceuticals during the COVID-19 outbreaks. On the other hand, it was surprising to find out that that only 0.3% of FGDs in the Singida region confirmed the use of traditional medicines. This finding reflects the disparities between regions in the way population utilised traditional medicines during the COVID pandemic.

### Lessons learnt from traditional medical practices as an alternative to COVID-19 vaccines

#### The role of indigenous knowledge in promoting traditional medicines during COVID-19 outbreak

The IK played an important role in promoting traditional medicines during the early days of the COVID-19 pandemic. The IK related to the treatment of common colds and flu provided the reasons for people to confidently use traditional medicines to treat symptoms related to COVID-19. For many tribes in Tanzania, steam inhalation has existed since time immemorial and is one of the traditional methods of treating some ailments, including flu. Therefore, despite the political support, IK played a major role in awaking the people to lean towards traditional medicines during the early days of the outbreak. Some assertions from KII include:

‘Herbal steam inhalation has been part of our traditional practices since time immemorial. When a family member falls sick does steam inhalation, and sometime when one has fever, we take hot water in the bucket and bring a patient close then cover him/her with a blanket. When a patient sweats then we know the fever is gone.’ (KII 05M, Mbeya CC, Mbeya)

‘If the patients lived closer to the health facility, e.g. a dispensary or health centre, they were taken to the health facility to get treated. However, there were those home-based interventions which are based on traditional medicines or alternative medicines. We have been doing herbal steam inhalation, and taking mixture of ginger, lemon, garlic, and onions. During COVID-19 outbreak, these traditional medicines and nutraceuticals were used so much as interventions, and they were effective.’ (KII 02F, Singida DC)

### Massive promotion of COVID-19 vaccines in Tanzania at the backdrop of the strengthened traditional medical practices

Tanzania experienced two different paradigms in the response against COVID-19. The first paradigm was driven by the lack of modern medicine to tackle the COVID-19 outbreak during the early days of the pandemic. With limited or no options, the government promoted traditional medicines as a safe, effective and reliable solution. The promotion was based on the history of use or IK on safety and efficacy. Despite the increasing regulatory questions on traditional medicines, the strategy succeeded in making the public believe this was the solution, hence helping to quell fear and panic among people. As the traditional medicine practices enjoyed support, it was during the same time that doubt and suspicions on the COVID-19 modern therapeutics were increasing.^18^ The demise of President John Magufuli in March 2021 marked the beginning of the new or second paradigm on the response against COVID-19. Under the new presidency of HE Samia Suluhu Hasan, science was given its deserved space to lead the process of deciding which intervention would put the country in the best position to fight incoming waves of COVID-19 outbreaks. This was the moment political pronouncement about COVID-19 vaccines changed by beginning to promote information about modern medicines. The government began to advocate for COVID vaccines as safe, reliable and efficacious interventions that should be introduced in the country as a matter of urgency.^13^

To efficiently disseminate information about COVID-19 vaccines to the Tanzanian population, political, social and public health approaches backed by a massive media promotion were needed. Consequently, massive health promotion activities on COVID-19 vaccines were conducted to dispel myth, fear and negative rumours in the communities. Different players from the national to village level were involved in the dissemination of positive messages about COVID-19 vaccines across the country. This was also revealed during the KII and FGD engagements in the eight regions. Some of the attestations are provided further in the text:

‘Information about COVID-19 vaccines were shared through television and radio announcements. For instance, we heard the Minister of Health, Honourable Ummy Mwalimu and HE President Samia Suluhu promoting and urging people to get vaccinated. In other words, this required leaders with a strong convincing power to help people understand the messages and get vaccinated.’ (KII 03M, Morogoro DC, Morogoro)

‘We were providing health education about the vaccines in churches, village meetings, and weekly village markets and auctions. During these meetings we also explained about safety and efficacy of the vaccines, and why it is important for people to get vaccinated.’ (KII 01F-HCW, Chunya DC, Mbeya)

It was at this stage that the adoption or use of the health belief model^20,21^ was necessary to enable the population to better understand the concept of preventive medicine and its benefits.

The push messages or SMS on the perceived susceptibility to COVID-19 infections were developed particularly when the news about the new wave of outbreak was constantly streaming in. The news coupled with the worst experience of the Delta strain, which circulated in Tanzania from December 2020 to May 2021, made it possible for the population to pay attention to the new call for the uptake of COVID-19 vaccines.

Additionally, the messages on the benefits of getting vaccinated against COVID-19 virus created a better understanding of the safety and benefit of vaccines. As one of the key informants asserted:

‘The Ministry of Health has done a better job as the deployed public announcers with their powerful speakers who passed through streets, and made similar announcement in the churches and on Saturdays, they were at the marketplaces promoting COVID-19 vaccines. They provided a better understanding on how it will protect us from another potential outbreak. Furthermore Nurses from Tengeru Hospital managed to reach out to huge section of the population to promote the vaccine. Truly, nurses did a great work in ensuring that many people accept COVID-19 vaccines.’ (KII 05F, Meru DC Arusha)

One of the benefits well-disseminated during the promotion of COVID-19 vaccines was the creation of a national herd immunity driven by mass immunisation, thus impeding the circulation of the virus in the community. This created a feeling that the vaccines are everyone’s business. On addressing barriers, the health promotion messages were designed to dispell negative rumours, myth and fear about the vaccines. This was more effective when HE President Samia Suluhu Hassan was publicly vaccinated on 28 July 2021 to prove to the nation that the vaccines are safe and efficacious. Thereafter, the demand for the wider access to vaccines increased, even though at that time the available stocks were for special groups. At this stage, the country was experiencing a complete paradigm on the response to COVID-19 pandemic. A holistic strategy in providing health education across the country yielded a positive impact, and the population was able to understand and thus heeded to the call to avail the COVID-19 vaccines as attested by KII and FGD participants:

‘The strategies used was a holistic strategy whereby national level, regional, and district leaders got out of their offices to promote COVID-19 vaccines. Some of these leaders went down to the wards and villages to promote vaccines. For instance, at our village the District Commissioner and District Administrative Secretary came. These key figures at the district level provided health education on COVID-19 vaccines and convinced many. There were several people at our village who got vaccinated on that very day of the campaign, while many other were vaccinated in the subsequent days.’ (KII 01M, Urambo DC, Tabora)

### Challenges of traditional medicine practices as alternatives to COVID-19 vaccines

#### Proliferation of unregulated herbal medicines formulated for COVID-19

The political promotion of traditional medicines received an overwhelming response from the citizens. This resulted in the massive development of unregulated herbal formulations, which exposed the populations to health risks like mid- and long-term toxicity issues. Given the level of consumption and the urgency to protect oneself against COVID-19, majority of the people could not bother to question the safety and efficacy of the formulations traded in their communities.

The development of NIMRCAF by the NIMR brought relief to the population and enhanced the trust of people on local solutions. But this triggered more interests from traders and herbalists to develop products that were sold at higher prices. Therefore, despite the positive response and contribution of traditional medicines in calming down the fear, the prices at which most of the herbal formulations traded at, was beyond the reach of most of the people. The cost of accessing branded herbal formulations circulating in the market triggered proliferation of homemade herbal medicines, as per the following KII assertion:

‘It means this herbal steam inhalation is just a normal for us, ehee, therefore this is very meaningful as even HE President urged citizen to do steam inhalation using different types of leaves and barks as per their traditions. Today I can tell you that am doing herbal steam inhalation using leaves of neem tree, or banana plant, or any medicinal plant available in our setting and we know it can be used for that purpose.’ (KII 02M, Mtwara DC, Mtwara)

‘They prepare a mixture of lemon and ginger, this helped many people, as many mostly used this mixture. Many people did not go to hospital as they just resorted to self-treatment using a concoction of lemon, ginger, garlic etc, and they further used traditional medicines as they considered COVID-19 as like flu.’ (KII 04M, Mtwara DC, Mtwara)

It was further evident that a trust on traditional medicines versus COVID-19 vaccines grew and this had a negative impact on the uptake of the vaccines at the early days of introduction. Some of the assertions from FGD participants include:

‘… Probably they should use alternative medicines, but saying that we should go for COVID-19 vaccines aaah aah no. I still have some doubts and am scared.’ (FGD 03, P09F, Mbeya CC, Mbeya)

### Indigenous knowledge versus modern medicine

This study has revealed that IK has a huge influence on health-seeking behaviour in most communities. In the rural areas, particularly pastoralist communities, traditional medicines have been taking the lead ahead of modern medicines. Therefore, the political influence and directives on the use of traditional medicines were met by the beliefs and practices, which existed long before the COVID-19 outbreak. An example of this scenario are the Maasai people, who said the following:

‘We normally take patients to the hospital or a health centre. But for us Maasai people we first try herbal medicines, and steam inhalation is one of the first steps in the treatment process. There is no Maasai who take a patient directly to the hospital before trying traditional practices. We do try this and that, and if all traditional practices fail then we take a patient to hospital.’ (FGD 04, P011M, Arusha CC, Arusha)

Another assertion on this aspect came from a ‘Laigwanani’, a Maasai leader, who said:

‘We take patients to the hospital, but should the modern medicines fail we take the patient to the forest for traditional medicine. There we slaughter a ship and use some herbs to cure the disease. We have taken many patients to the forest, treated them with our traditional medicines and they get cured. therefore, many don’t go to the hospital. Haven’t you heard what HE President Magufuli said?’ (KII 06M, Arusha DC-Oldonyo Sambu, Arusha)

Like the Maasai people, the communities in the Southeastern region of Mtwara have had the culture of using steam inhalation for generations. Thus, a promotion of using steam inhalation as one of the traditional medical practices to treat COVID-19 symptoms was just a reminder to uphold their traditional practices. One of the key informants asserted:

‘For us steam inhalation is a normal practice. Even when HE President Magufuli called for people to use various traditional medicines including steam inhalation, for us it was just an emphasis on what we are practicing regularly. We use leaves and stembarks from different plant species. For instance, we use neem or banana tree leaves, while in some places they use other plants for the same purposes.’ (KII 01F, Mtwara DC, Mtwara)

### Political influence on traditional medicines and a change of narrative on COVID-19 vaccines

When the first case of COVID-19 was announced in Tanzania, the world had no clues on the recommended medical intervention, which could be used in the treatment and management of COVID cases. This void was filled by the government call for action from different knowledge domains, including religions and traditional medicines, to work towards finding solutions for COVID-19. It was during this period when fear of the virus was so huge that some healthcare workers were hesitant to attend to patients who presented at the health facilities with COVID-19-related symptoms. During the KII, some informants indicated that the negative attitude of healthcare workers scared people away; therefore, they resorted to traditional medicines as an available and friendly medical attention. Some of these experiences were provided by the key informants, for instance:

‘Whether it is corona or not corona, Doctors at the health facilities were so scared at the beginning. When you tell him/her that you have corona, they ran away and/or refusing to provide care to the patient. So where do you could we get information about the diseases, from media?’ (KII 03M, Chunya DC, Mbeya)

Despite the fear of the disease among healthcare workers experienced in some parts of the country, THPs were bold enough to accommodate patients who sought care and treatment at their point of service delivery. This enhanced trust on traditional medicines and hence any medications prescribed by the THPs were easily accepted and used by patients. With a growing political influence, the use of traditional medicines over modern medicines grew and was deeply entrenched in the communities. Thus, shifting the minds and trust of the people from this domain to embrace COVID-19 vaccines needed extra efforts. A few quotes from KIIs and FGDs attest to the level of trust people had on traditional medicines and how this contributed to COVID-19 vaccines’ hesitancy:

‘I have not been vaccinated for two reasons, first the government leadership particularly the head of state at that time HE President Magufuli was against COVID-19 vaccines and insisted on thorough investigation before any attempt to introduce them in the country. However, when HE Samia Suluhu Hassan took over leadership there was an abrupt change of narrative, she promoted COVID-19 vaccines. A question was why such a sudden change of mind while a few weeks ago when she was a Deputy President could not advise President Magufuli about Vaccines? This is my main concern, why now allowing the vaccines? This is what has made me and several other not to get vaccinated.’ (FGD 03, P10M, Morogoro DC, Morogoro)

‘… At our area there was confusion because, a change of Head of State brought in a completely different approach. Initially, the late President John P. Magufuli was not pro-vaccines, he promoted traditional medicines and locally made solutions. His successor President Samia Suluhu Hassan came up with the COVID-19 vaccines agenda. Till today in our community majority of the people believe on the first approach while some are supporting the current approach on COVID-19 vaccines. Already, you can see it was a difficult to accept the vaccines because, many believe that during the first year of the pandemic the government managed the outbreak well, and the treatment options worked well. Fortunately, when President Samia got vaccinated in public, it helped to change the thinking of many and we started asking ourselves, why can’t I also get my jab?’ (KII 05F, Njombe TC, Njombe)

## Discussion

The contagious nature of the COVID-19 virus created shock, fear and panic in the communities, decision-makers and healthcare service providers. The first wave of the outbreak found most countries unprepared, which compelled governments and communities to jointly find local solutions that would reduce the severity and negative outcomes of the disease. Tanzania’s experience is intriguing because the country adopted two different response approaches determined by political directions. As the whole world was working hard to get the possible interventions, which would protect the public from COVID-19 infection, Tanzania had to turn to IK where traditional medical practices were sought to treat the infection.

Driven by both politics and IK, a call was made to citizens to look for local remedies, which will help in treating the symptoms associated with COVID-19. A massive campaign led by the government itself promoted the use of traditional medical practices as part of the response to the pandemic. In one of the public addresses held on 11 June 2020, HE President Magufuli said:

For sometimes now we have been cowards of using our traditional and alternative medicines and practices due to developments in science and technology. We must change! Conventional medicines come from herbs that are referred as traditional and alternative medicines.^9^

As revealed during KII and FGDs, the call received a massive response, and various traditional medical practices, such as steam inhalation, drinking concoctions made from different plant species, and inhaling infused essential oils, were used, leading to the development of some products. On the other hand, herbalists and entrepreneurs developed products such as ‘covidol, covotanxa, plante++, uzima, bupiji, shengena, ronah, shekilindi, bycaro, and bingwa’, to mention a few,^9^ which were introduced in the market. Despite limited or lack of data on the safety and efficacy of these products, the public consumed the products.

The KII and FGDs have revealed that many members of the communities resorted to preparing their anti-COVID products at home. The preparations were informed by shared information through social media, government official’s pronouncements and an overall promotion made on traditional medicines. Interestingly, the political promotion of traditional medicines was met by the existing IK, making it easy for the messages to penetrate in the minds of the people. Consequently, the messages reached to the far ends of the country in such a way that communities, which were not practicing steam inhalation before, started to practice it as a curative and preventive strategy against COVID-19.

There are several lessons that we can learn from the practice of traditional medicines as an alternative intervention during the early days of the COVID-19 pandemic. Firstly, it is important to consider IK during health emergencies; however, the practices must be well-regulated to protect the public from potential harm. Secondly, countries need to consider THPs in the preparedness, prevention and response against health emergencies, as they have a huge following in the communities and can easily be reached. This was confirmed by participants from all the eight regions; no participants challenged traditional medical practices used during the COVID-19 outbreaks. Thirdly, there must be deliberate investment in research on traditional and alternative medicines so that scientific evidence on the products should be generated to support their use. Learning from Traditional Chinese Medicines (TCM) there have been several scientific reports supporting the use of TCM against the COVID-19 pandemic.^21,22^ Tanzania and other African countries need to invest on empirical research on traditional medicines to support their use, particularly during health emergencies. Lastly, political influence is a big factor in disseminating information and pulling the public to lean towards certain interventions. This was the case for Tanzania where politicians promoted traditional medicines use, and it helped to quell fear, shock and panic brought by the COVID-19 pandemic.

This study has further provided a lesson on how impactful political influence can be in promoting one aspect of an intervention against the other. For example, in Tanzania, there was an emphasis on the use of traditional medicines while casting doubts about modern medicines in public. This created a challenge when the country turned to modern medicine by starting to promote COVID-19 vaccines. Consequently, massive resources had to be used to conduct a countrywide health promotion to enable the population to reasonably understand the message and the change of political direction towards COVID-19 interventions. Even though the campaign succeeded, it left behind a lesson that public figures’ pessimism against certain interventions should be handled carefully.

## Conclusion

Political influence and IK had a huge contribution towards public leaning on traditional medicines during the early days of the COVID-19 pandemic in Tanzania. While the public used traditional medicines, doubts on the safety and efficacy of COVID-19 vaccines were increasing and fuelled by political utterances. However, the study has revealed that a change of political direction coupled with a massive health promotion across the country managed to break the COVID-19 vaccine uptake hesitancy. Many Tanzanians across the country got vaccinated. The Tanzanian scenario provides several lessons; included among them are the need to integrate science and traditional medical practices to generate evidence, which guide response during health emergencies; political direction and influence during disease outbreaks should be informed by scientific evidence to avoid public confusions; IK though important should be used carefully to ensure the safety of the public. Having experienced two different paradigms on responding to COVID-19, Tanzania provides a learning experience, which will inform best response strategy in the future epidemics.
